# Unraveling the Distinct Roles of Al and Ca in Microstructure Evolution and Tensile Response of Extruded Mg–Al–Ca Alloys

**DOI:** 10.3390/ma19122638

**Published:** 2026-06-18

**Authors:** Chen Chen, Junbo Wang, Yong Wang, Changyu Hu, Shengxiong Tang, Ranfeng Qiu, Yiwen Chen

**Affiliations:** 1 School of Intelligent Manufacturing, Jiangnan University, Wuxi 214122, China; 2 School of Materials Science and Engineering, Harbin Institute of Technology, Harbin 150001, China; 3 Department of Materials Science, Graduate School of Science and Technology, Kumamoto University, 2-39-1 Kurokami, Chuo-ku, Kumamoto 860-8555, Japan; 4 School of Materials Science and Engineering, Henan University of Science and Technology, Luoyang 471039, China

**Keywords:** Mg–Al–Ca alloy, fine Mg_17_Al_12_ particles, grain refinement, Orowan strengthening, mechanical properties

## Abstract

Mg-Al-Ca alloys are attractive low-cost wrought Mg alloys. However, the distinct roles of Al and Ca in regulating deformation-processed microstructures and mechanical properties remain unclear. In this work, Mg–6Al–0.5Ca, Mg–9Al–0.5Ca, and Mg–9Al–1.3Ca (wt. %) alloys were extruded at 250 °C and 300 °C to clarify the composition-dependent microstructure evolution and strengthening mechanisms. Increasing the Al content from 6 to 9 wt. % markedly promoted the formation of fine Mg_17_Al_12_ (f-Mg_17_Al_12_) and coarse Mg_17_Al_12_ particles, whereas increasing the Ca content from 0.5 to 1.3 wt. % promoted the formation of coarse Al_2_Ca particles while reducing the density of f-Mg_17_Al_12_. Quantitative analysis revealed that f-Mg_17_Al_12_ particles refined dynamically recrystallized grains by promoting recrystallization nucleation and pinning grain boundaries while also contributing to Orowan strengthening. The Mg–9Al–0.5Ca alloy exhibited the best strength–ductility balance, with a yield strength of 338 ± 4 MPa, ultimate tensile strength of 396 ± 5 MPa, and elongation of 8.7 ± 1.6% after extrusion at 250 °C. Strengthening calculations indicated that grain-boundary strengthening was the dominant strengthening contribution, while the strength advantage of Mg–9Al–0.5Ca originated from the dual role of f-Mg_17_Al_12_ in grain refinement and dislocation obstruction. These findings provide a practical strategy for designing high-strength non-rare-earth Mg–Al–Ca extrusion alloys.

## 1. Introduction

Mg-Al-based magnesium alloys are among the most widely used low-cost Mg alloy systems because of their good castability, relatively high specific strength, and mature processing routes [1,2,3,4,5,6]. However, their broader engineering application is still restricted by their limited thermal stability and insufficient mechanical performance. This limited thermal stability is related to the instability and coarsening tendency of β-Mg_17_Al_12_ at elevated temperatures [7,8]. Therefore, considerable efforts have been devoted to modifying conventional Mg-Al alloys through alloy design to improve both their mechanical properties and deformation capability [9].

Ca is widely regarded as an effective alloying element in Mg-Al alloys because it suppresses the formation of β-Mg_17_Al_12_ and promotes the formation of thermally stable Ca-containing intermetallic phases, such as Al_2_Ca with a C15 or C36 Laves structure [10,11,12,13,14]. The Al_2_Ca phase is often beneficial for yield stress (YS) and heat resistance because it stabilizes the microstructure and hinders dislocation motion during deformation [11,12,15]. At the same time, Ca also affects deformation behavior after thermomechanical processing by altering second-phase fragmentation, recrystallization, and texture development [15,16]. For example, Zubair et al. [12] investigated three alloys, namely, Mg-6Al-2Ca, Mg-5Al-3Ca, and Mg-4Al-4Ca, and showed that the strengthening effect of the Laves phase depends strongly on its crystal structure and spatial distribution rather than simply on the Ca content itself. Li et al. found that the Ca/Al ratio in extruded Mg–Al–Ca–Mn alloys strongly influenced the tensile properties, recrystallization behavior, and texture intensity [15]. Nevertheless, Ca is not universally beneficial. When its content becomes excessive, coarse or brittle intermetallic compounds can form and act as crack initiation sites, thereby impairing tensile deformation and reducing formability [12,16]. This indicates that the contribution of Ca to mechanical performance depends not only on its content but also on how it modifies the phase constitution after processing.

Compared with Ca, Al plays a more direct and complex role in determining the final response of Mg-Al-Ca alloys. In addition to providing solid-solution strengthening to the Mg matrix, Al strongly affects the partitioning of Ca into secondary phases and therefore influences both the amount of strengthening phases and the way by which strain is accommodated during deformation [17,18]. Chai et al. [17] reported that varying the Al content in extruded Mg-3.5Ca alloys led to pronounced changes in tensile strength, ductility, and deformation texture. Li et al. [18] also found that the Al content in Mg-Al-Ca-Mn extrusion alloys significantly altered the eutectic phase composition, recrystallized microstructure, and resulting strength–ductility balance. In dilute Mg-Al-Ca-Mn alloys, Nakata et al. [19,20] showed that suitable Al additions, together with optimized Ca and Mn contents, enabled both high-speed extrusion and subsequent age hardening. Similar findings have also been reported for sheet alloys, in which properly designed Al- and Ca-containing compositions exhibited both age hardenability and good room-temperature formability [21,22]. These findings suggest that the roles of Al and Ca are closely coupled and that the final properties of Mg–Al–Ca alloys are governed by their combined effects on strengthening, strain accommodation, and deformation-processed microstructures.

Despite these advances, previous studies have mainly focused on high-Ca heat-resistant cast alloys or dilute Mg–Al–Ca–Mn wrought alloys designed for rapid extrusion, age hardening, or sheet formability [19,23,24]. In comparison, the roles of Al and Ca in Mg–Al–Ca alloys with moderate Ca contents remain insufficiently clarified. In particular, under comparable extrusion conditions, it remains unclear whether increasing Ca and increasing Al affect tensile properties through similar mechanisms or lead to fundamentally different responses in strengthening, ductility, and deformation behavior [15,17,18]. This issue becomes more critical when the extrusion temperature varies because the competition among second-phase fragmentation, dynamic recrystallization, and strain accommodation is highly sensitive to both alloy chemistry and processing temperature [25].

In the present work, three Mg–Al–Ca alloys, Mg–9Al–0.5Ca (wt. %, AX90), Mg–9Al–1.3Ca (wt. %, AX91), and Mg–6Al–0.5Ca (wt. %, AX60), were designed to separately evaluate the effects of Ca and Al contents. These alloys were extruded at 250 °C and 300 °C under the same ram speed and extrusion ratio, and their microstructures and tensile properties were systematically characterized using OM, SEM, EBSD, TEM, and room-temperature tensile testing. This study aimed to clarify how Al and Ca differently regulate strengthening, deformation behavior, and deformation-processed microstructures in extruded Mg–Al–Ca alloys.

## 2. Materials and Methods

Three Mg-Al-Ca alloys with nominal compositions of AX90, AX91, and AX60 were prepared by conventional melting and casting. High-purity Mg (99.95 wt. %), Al (99.999 wt. %), and Ca (99.99 wt. %) were used as raw materials. Alloy melting was carried out in a resistance furnace under a CO_2_/SF_6_ protective atmosphere to minimize oxidation and burning loss. The pure Mg was first melted at approximately 720 °C, followed by the addition of Al and Ca into the melt. After all alloying elements were fully melted, the melt was held at 720 °C for 20 min and mechanically stirred to improve compositional homogeneity. The melt was then cooled to approximately 700 °C and poured into a preheated steel mold to obtain cast ingots. The precise composition and levels of impurity elements (Fe, Cu, Ni, Si, etc.) of the AX90, AX91, and AX60 alloys were quantified using inductively coupled plasma atomic emission spectroscopy (ICP-AES, Thermo Fisher Scientific, Waltham, MA, USA), as listed in Table 1. The cast ingots were cylindrical rods with an initial diameter of 60 mm and a length of 900 mm. After casting, the ingots were homogenized at 400 °C for 8 h, followed by water quenching. Subsequently, all homogenized ingots were extruded at 250 °C and 300 °C, respectively, with a constant ram speed of 1.0 mm/s and an extrusion ratio of 12:1. The extruded alloys are hereafter designated as AX60-EX250, AX90-EX250, AX91-EX250, AX60-EX300, AX90-EX300, and AX91-EX300, according to their compositions and extrusion temperatures.

The microstructures of the as-extruded alloys were first examined by optical microscopy (OM). Metallographic specimens were cut from the extruded alloys along the extrusion direction (ED). After grinding with SiC abrasive papers, the specimens were polished using a MgO suspension and then etched for 1 min with a solution consisting of 10 mL acetic acid, 10 mL distilled water, 4.2 g picric acid, and 70 mL ethanol for OM observation. Phase identification was performed using a D8 Advance X-ray diffractometer (XRD, Bruker, Germany) with Cu K_α_ radiation (λ = 1.5406 Å). Phase constitution and microstructural features were further characterized using scanning electron microscopy (SEM, HITACHI S-4800, Hitachi High-Tech Corporation, Ibaraki, Japan) equipped with backscattered electron (BSE) imaging, energy-dispersive spectroscopy (EDS), and electron backscatter diffraction (EBSD) detectors. The specimens used for SEM observation were the same as those prepared for OM. Prior to the EBSD measurements, the specimens were mechanically polished and then electrochemically polished in a solution of 10 vol.% perchloric acid applied at 30 V for 2 min in ethanol to obtain deformation-free surfaces. Data including inverse pole figure (IPF) maps, dynamic recrystallization (DRX) grain size distributions, and pole figure (PF) images were processed using Orientation Imaging Microscopy (OIM) Analysis software (version 8.6, EDAX LLC, Mahwah, NJ, USA).

Room-temperature tensile tests were carried out to evaluate the mechanical properties of the AX90, AX91, and AX60 alloys. Dog-bone-shaped tensile specimens with a gauge size of 18 mm × 1.5 mm were machined from the extruded rods, with the tensile axis parallel to the ED. Tensile testing was performed using a universal testing machine at a constant initial strain rate of 5 × 10^−4^ s^−1^. For each condition, at least three specimens were tested to ensure reproducibility.

## 3. Results

### 3.1. Microstructural Evolution of As-Cast and Homogenized States

**Figure 1:**
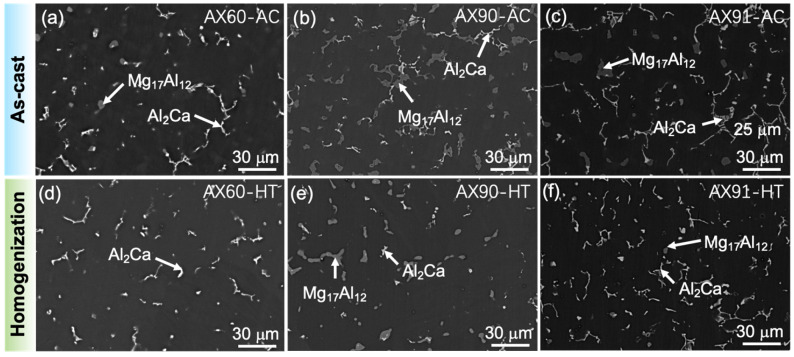
BSE images of the as-cast and homogenized Mg-Al-Ca alloys: (**a**,**d**) AX60, (**b**,**e**) AX90, and (**c**,**f**) AX91. The as-cast microstructures are shown in (**a**–**c**), while the homogenized microstructures are shown in (**d**–**f**). The Mg_17_Al_12_ and Al_2_Ca phases are indicated by arrows.

BSE images of the as-cast and homogenized Mg-Al-Ca alloys are shown in Figure 1. In the as-cast condition, all three alloys exhibit typical α-Mg matrices with interdendritic second phases. In AX60-AC, a small amount of Mg_17_Al_12_ and Al_2_Ca phases is discontinuously distributed along the interdendritic regions. With increasing Al content, AX90-AC contains a larger amount of Mg_17_Al_12_, while Al_2_Ca particles are also observed. For AX91-AC, the higher Ca content promotes the formation of more Al_2_Ca phases, and the second phases become more pronounced and locally connected. After homogenization at 400 °C for 8 h, the continuous or semi-continuous second-phase networks are largely broken and transformed into more discrete particles. In AX60-HT and AX90-HT, the Mg_17_Al_12_ phase is reduced and redistributed, while Al_2_Ca particles remain because of their relatively high thermal stability. In AX91-HT, a higher number density of Al_2_Ca particles is still retained compared with AX60-HT and AX90-HT. These results indicate that homogenization reduces the segregation and continuity of the as-cast second phases, but the phase constitution and particle distribution remain strongly dependent on the Al and Ca contents.

### 3.2. Microstructural Evolution After Extrusion at 250 °C

**Figure 2:**
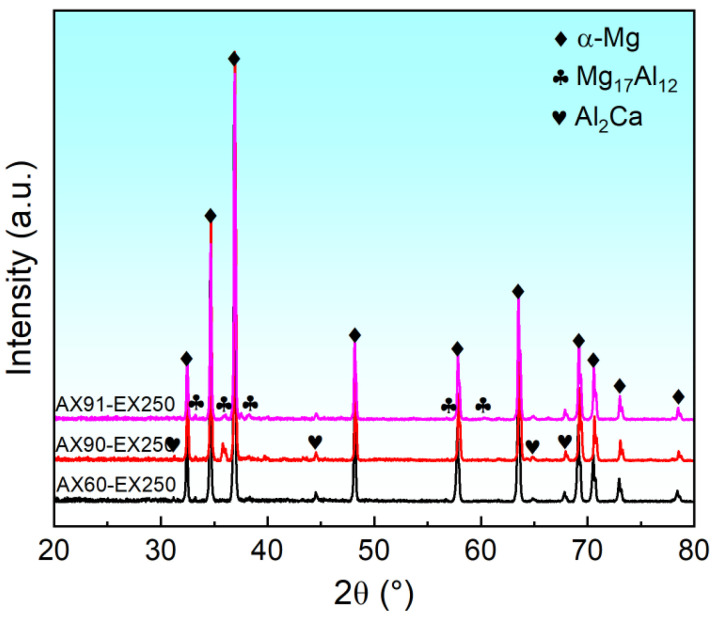
XRD patterns of AX60-EX250, AX90-EX250, and AX91-EX250 alloys.

The XRD patterns of the alloys extruded at 250 °C are shown in Figure 2. AX60-EX250 is mainly composed of α-Mg, Mg_17_Al_12_, and Al_2_Ca phases. AX90-EX250 also consists of α-Mg, Mg_17_Al_12_, and Al_2_Ca phases, with clearly detected Mg_17_Al_12_ diffraction peaks. For AX91-EX250, α-Mg, Mg_17_Al_12_, and Al_2_Ca phases are identified, and the Al_2_Ca phase is also detected owing to the higher Ca content. These results confirm that the extruded Mg-Al-Ca alloys contain an α-Mg matrix together with Mg_17_Al_12_ and Al_2_Ca intermetallic phases.

**Figure 3:**
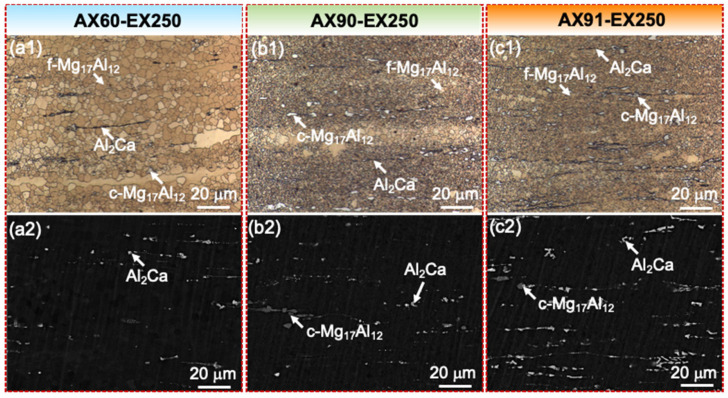
OM and SEM-BSE images of the extruded alloys at 250 °C: (**a1**,**a2**) AX60-EX250, (**b1**,**b2**) AX90-EX250, and (**c1**,**c2**) AX91-EX250. The OM images in (**a1**–**c1**) and the BSE images in (**a2**–**c2**) show the deformation-processed microstructures parallel to the ED. The fine Mg_17_Al_12_ phase (f-Mg_17_Al_12_), coarse Mg_17_Al_12_ phase (c-Mg_17_Al_12_), and Al_2_Ca phase are indicated by arrows.

Figure 3 shows OM and BSE images of AX60-EX250, AX90-EX250, and AX91-EX250 observed parallel to the ED. After extrusion at 250 °C, all three alloys exhibited typical banded deformation-processed microstructures, in which the second phases were elongated, fragmented, and redistributed along the ED. In AX60-EX250, the microstructure consisted of a relatively coarse Mg matrix and a small amount of second phases, as shown in Figure 3(a1,a2). Fine Mg_17_Al_12_ (f-Mg_17_Al_12_) particles were dispersed in the matrix, while coarse Mg_17_Al_12_ (c-Mg_17_Al_12_) and Al_2_Ca phases were mainly distributed as discontinuous bands along the ED. Compared with AX60-EX250, the AX90-EX250 alloy showed a more pronounced banded structure and a higher number density of f-Mg_17_Al_12_ particles, as shown in Figure 3(b1,b2). c-Mg_17_Al_12_ and Al_2_Ca phases were also observed in AX90-EX250, but they were more discontinuously distributed after extrusion, indicating stronger deformation-induced redistribution of the second phases. In AX91-EX250, the banded morphology remained evident, and the amounts of c-Mg_17_Al_12_ and Al_2_Ca phases were higher than those in AX60-EX250 and AX90-EX250, as shown in Figure 3(c1,c2). These coarse second phases were preferentially aligned along the ED and formed more obvious second-phase stringers. f-Mg_17_Al_12_ particles were still present in the matrix of AX91-EX250, but the microstructure was characterized by a larger fraction of coarse second phases than the other two alloys.

**Figure 4:**
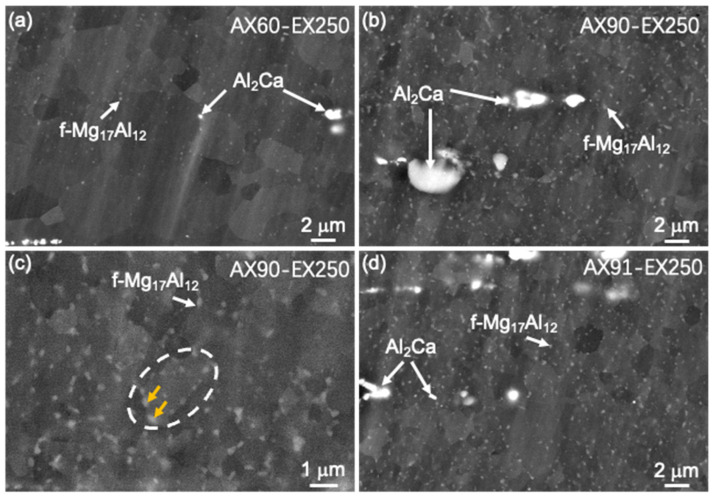
High-magnification SEM-BSE images of the extruded alloys at 250 °C: (**a**) AX60-EX250, (**b**,**c**) AX90-EX250, and (**d**) AX91-EX250. The dashed circle in (**c**) highlights the pinning effect of f-Mg_17_Al_12_ particles on a recrystallized grain boundary.

Figure 4 shows high-magnification BSE images of the second-phase particles in the alloys extruded at 250 °C. In AX60-EX250, only a small number of f-Mg_17_Al_12_ particles were observed in the matrix, whereas brighter Al_2_Ca particles could be distinguished by their higher contrast, as shown in Figure 4a. With increasing Al content, AX90-EX250 exhibited a markedly higher number density of fine particles both within grains and along grain boundaries (Figure 4b,c). Considering the higher Al content and the contrast difference from Al_2_Ca, these fine particles were identified as f-Mg_17_Al_12_. Some f-Mg_17_Al_12_ particles were located at recrystallized grain boundaries, as highlighted by the dashed circle in Figure 4c, indicating their effective potential pinning effect on boundary migration. In AX91-EX250, f-Mg_17_Al_12_ particles were still present, while more bright Al_2_Ca particles were observed owning to the increased Ca content (as shown in Figure 4d).

**Figure 5:**
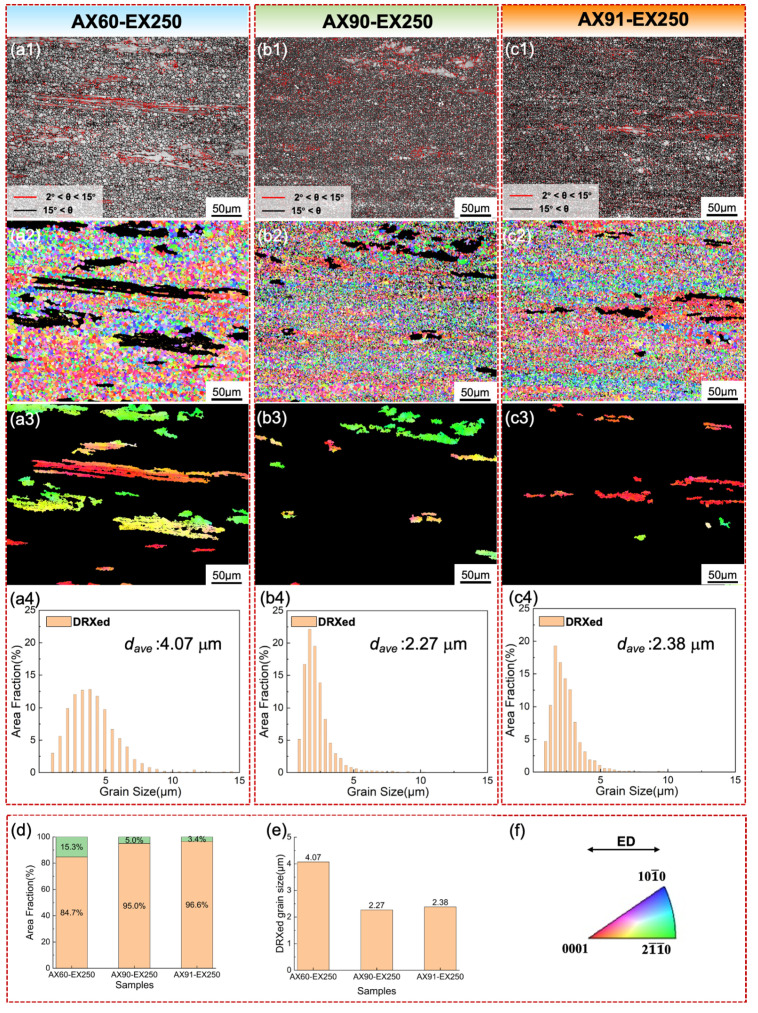
EBSD results of the extruded alloy at 250 °C: (**a1**–**c1**) band-contrast maps, where low-angle grain boundaries (2° < θ < 15°) and high-angle grain boundaries (θ > 15°) are marked by red and black lines, respectively; (**a2**–**c2**) inverse pole figure (IPF) maps for DRXed region; (**a3**–**c3**) IPF maps for non-DRXed region; and (**a4**–**c4**) the grain size distributions of DRXed grains for AX60-EX250, AX90-EX250, and AX91-EX250 alloys. (**d**) Area fractions of DRXed and unDRXed regions. (**e**) Average grain sizes of DRXed grains. (**f**) IPF color key for the ED.

EBSD characterizations of AX60-EX250, AX90-EX250, and AX91-EX250 are presented in Figure 5. The band-contrast maps overlaid with grain boundaries in Figure 5(a1–c1) show that all three alloys contained a large fraction of high-angle grain boundaries (θ > 15°) after extrusion at 250 °C, indicating extensive dynamic recrystallization. Low-angle grain boundaries (15° > θ > 2°) were mainly distributed in the elongated unDRXed regions. The extracted IPF maps of the DRXed grains in Figure 5(a2–c2) show relatively random crystallographic orientations, indicating that dynamic recrystallization weakened the deformation texture in the recrystallized regions. The DRXed grains in AX60-EX250 are relatively coarse, whereas those in AX90-EX250 and AX91-EX250 are much finer and more uniformly distributed. In contrast, the extracted maps of elongated unDRXed grains in Figure 5(a3–c3) show the remaining deformation bands along the extrusion direction, with AX60-EX250 retaining a larger fraction of elongated unDRXed regions than AX90-EX250 and AX91-EX250. The statistical results further confirm these observations. As summarized in Figure 5d, the DRXed area fraction increased from 84.7% in AX60-EX250 to 95.0% in AX90-EX250 and 96.6% in AX91-EX250. Meanwhile, the average DRXed grain size decreased from 4.07 μm in AX60-EX250 to 2.27 μm in AX90-EX250 and 2.38 μm in AX91-EX250, as shown in Figure 5e.

**Figure 6:**
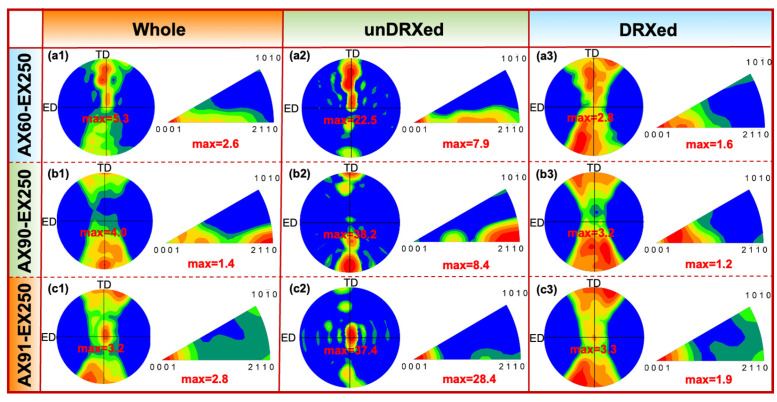
{0001} PFs and corresponding IPFs of the whole regions, unDRXed regions, and DRXed regions for the alloys extruded at 250 °C: (**a1**) whole region of AX60-EX250; (**a2**) unDRXed region of AX60-EX250; (**a3**) DRXed region of AX60-EX250; (**b1**) whole region of AX90-EX250; (**b2**) unDRXed region of AX90-EX250; (**b3**) DRXed region of AX90-EX250; (**c1**) whole region of AX91-EX250; (**c2**) unDRXed region of AX91-EX250; and (**c3**) DRXed region of AX91-EX250. Each subfigure contains a {0001} PF and the corresponding IPF. The maximum texture intensities are marked in each subfigure.

The {0001} PFs and IPFs of the whole, unDRXed, and DRXed regions of the alloys extruded at 250 °C are shown in Figure 6. The {0001} PFs reveal the basal texture characteristics of the three alloys. For the whole regions, AX60-EX250, AX90-EX250, and AX91-EX250 exhibited relatively weak basal textures, with maximum intensities of 5.3, 4.0, and 3.2, respectively, as shown in Figure 6(a1–c1). In contrast, the DRXed regions exhibited much weaker and more dispersed basal textures, with maximum intensities of 2.8, 3.2, and 3.3, respectively (Figure 6(a3–c3)). The corresponding IPFs show the texture distribution along the ED. The unDRXed regions displayed higher IPF intensities than the whole and DRXed regions, whereas the DRXed regions showed weaker and more scattered orientations. These results indicate that the strong deformation texture was mainly retained in the unDRXed regions, whereas dynamic recrystallization weakened and randomized the texture during extrusion at 250 °C.

### 3.3. Microstructural Evolution After Extrusion at 300 °C

**Figure 7:**
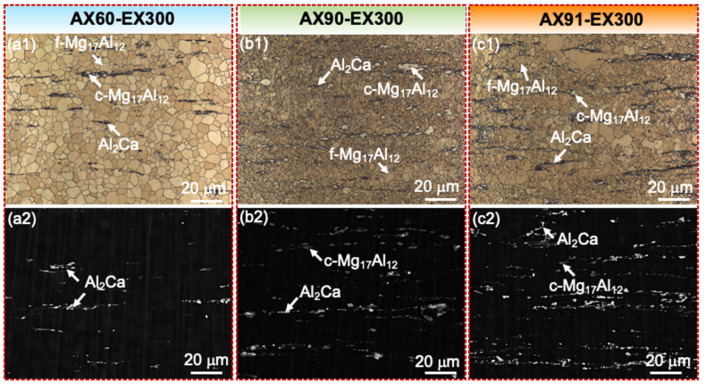
OM and BSE images of the extruded alloys at 300 °C: (**a1**,**a2**) AX60-EX300, (**b1**,**b2**) AX90-EX300, and (**c1**,**c2**) AX91-EX300. The OM images in (**a1**–**c1**) and BSE images in (**a2**–**c2**) show the deformation-processed microstructures parallel to the ED. The f-Mg_17_Al_12_, c-Mg_17_Al_12_, and Al_2_Ca phases are indicated by arrows.

After extrusion at 300 °C, the three alloys still exhibited banded deformation-processed microstructures along the ED, as shown in Figure 7. In AX60-EX300, the α-Mg grains were relatively coarse, and only a small amount of second phases was observed. The f-Mg_17_Al_12_, c-Mg_17_Al_12_, and Al_2_Ca phases were discontinuously distributed along the ED, as shown in Figure 7(a1,a2). In AX90-EX300, the banded structure became more evident, with a high density of f-Mg_17_Al_12_ particles in the matrix and discontinuously aligned c-Mg_17_Al_12_ and Al_2_Ca phases along the ED (Figure 7(b1,b2)). In AX91-EX300, more c-Mg_17_Al_12_ and Al_2_Ca phases were present and formed pronounced second-phase stringers along the ED, whereas the number density of f-Mg_17_Al_12_ particles was lower than that in AX90-EX300 (Figure 7(c1,c2)). Compared with the alloys extruded at 250 °C, the α-Mg grains after extrusion at 300 °C were generally coarser, especially in AX60-EX300 and AX91-EX300.

**Figure 8:**
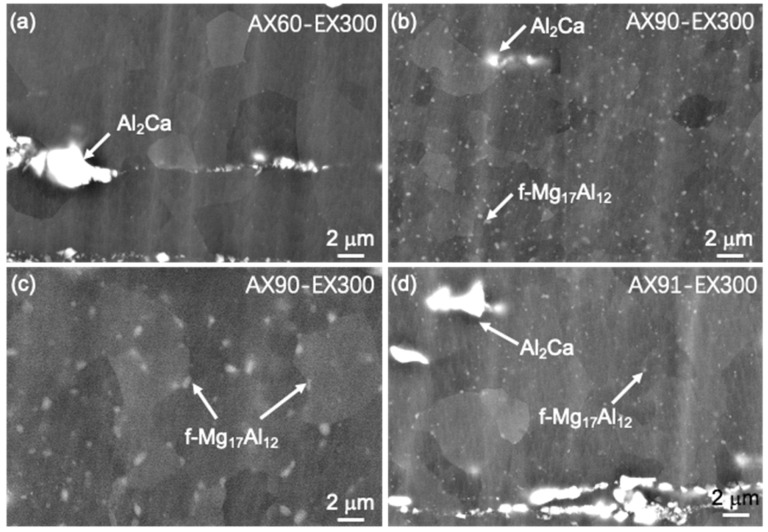
High-magnification BSE images of the extruded alloys at 300 °C: (**a**) AX60-EX300, (**b**,**c**) AX90-EX300, and (**d**) AX91-EX300. The f-Mg_17_Al_12_ and Al_2_Ca phases are indicated by arrows.

The high-magnification BSE images in Figure 8 further reveal the distribution of fine second-phase particles after extrusion at 300 °C. In AX60-EX300, f-Mg_17_Al_12_ particles were scarcely observed, and the visible bright particles corresponded to Al_2_Ca particles distributed along local second-phase bands (Figure 8a). In contrast, AX90-EX300 contained a high density of f-Mg_17_Al_12_ particles, which were distributed both within grains and near grain boundaries, as shown in Figure 8b,c. In AX91-EX300, f-Mg_17_Al_12_ particles were still present in the matrix, but their number density was lower than that in AX90-EX300, whereas brighter Al_2_Ca particles were observed owing to the higher Ca content (Figure 8d).

**Figure 9:**
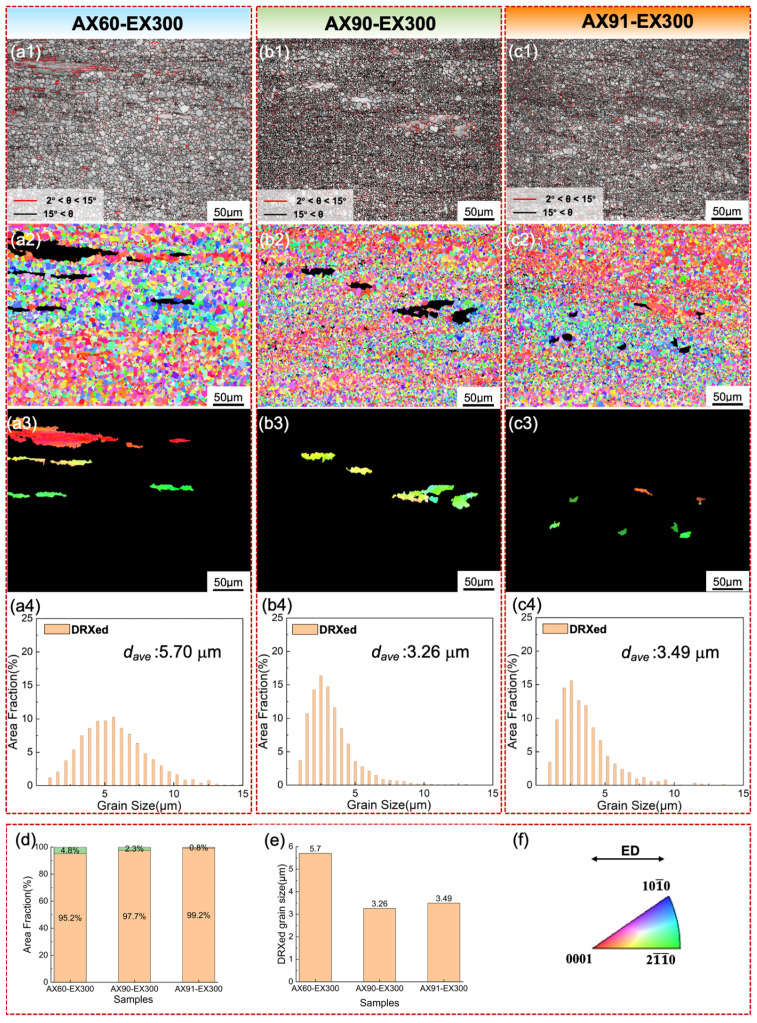
EBSD results of the alloy extruded at 300 °C: (**a1**–**c1**) band contrast maps overlapped with grain boundaries, where low-angle boundaries (2° < θ < 15°) and high-angle grain boundaries (θ > 15°) are marked by red and black lines, respectively; (**a2**–**c2**) extracted IPF maps of DRXed grains; (**a3**–**c3**) extracted maps of unDRXed regions; and (**a4**–**c4**) grain size distributions of DRXed grains for AX60-EX300, AX90-EX300, and AX91-EX300. (**d**) Area fractions of DRXed and unDRXed regions. (**e**) Average grain sizes of DRXed grains. (**f**) IPF color key for ED.

The EBSD results of the alloys extruded at 300 °C are presented in Figure 9. The band contrast maps overlapped with the grain boundaries in Figure 9(a1–c1) show that all three alloys were largely recrystallized, while a small fraction of elongated unDRXed regions was still retained along the ED. Compared with AX60-EX300, the elongated unDRXed regions were reduced in AX90-EX300 and AX91-EX300. The extracted IPF maps of the DRXed grains in Figure 9(a2–c2) show relatively random orientations and reveal clear differences in grain size among the three alloys. AX60-EX300 exhibited the coarsest DRXed grains, with an average grain size of 5.70 μm, whereas AX90-EX300 and AX91-EX300 showed much finer DRXed grains with average grain sizes of 3.26 μm and 3.49 μm, respectively. The extracted maps of the unDRXed regions in Figure 9(a3–c3) further confirm that AX60-EX300 contained more residual elongated deformed grains than the other two alloys. As summarized in Figure 9d, the DRXed area fraction increased from 95.2% in AX60-EX300 to 97.7% in AX90-EX300 and 99.2% in AX91-EX300. The corresponding average DRXed grain sizes are compared in Figure 9e, further showing the finer recrystallized microstructures in AX90-EX300 and AX91-EX300.

**Figure 10:**
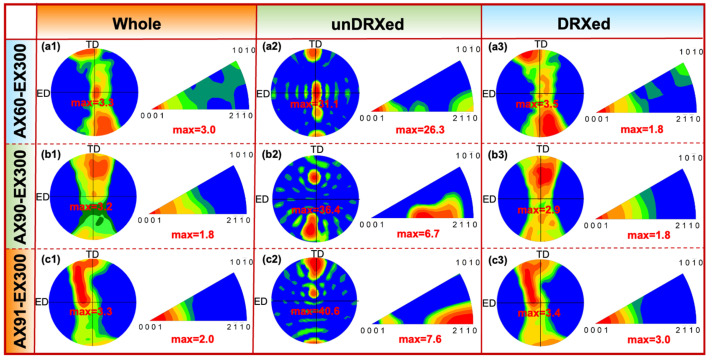
{0001} PFs and IPFs of the whole regions, unDRXed regions, and DRXed regions for the alloys extruded at 300 °C: (**a1**–**a3**) AX60-EX250, (**b1**–**b3**) AX90-EX250, and (**c1**–**c3**) AX91-EX250. The maximum texture intensities are marked in each figure.

The {0001} PFs and IPFs of the alloys extruded at 300 °C are shown in Figure 10. For the whole regions, AX60-EX300, AX90-EX300, and AX91-EX300 exhibited relatively weak basal textures, with maximum {0001} PF intensities of 3.3, 3.2, and 3.3, respectively (Figure 10(a1–c1)). After separating the unDRXed regions, the basal texture became much stronger, with maximum intensities of 21.1, 36.4, and 40.6 for AX60-EX300, AX90-EX300, and AX91-EX300, respectively (Figure 10(a2–c2)). In contrast, the DRXed regions showed much weaker basal textures, with maximum intensities of 3.5, 2.9, and 3.4, respectively (Figure 10(a3–c3)). The IPFs also show a clear difference between the unDRXed and DRXed regions. The unDRXed regions exhibited stronger and more concentrated orientations along the ED, whereas the DRXed regions displayed weaker and more dispersed orientations. This indicates that the strong deformation texture was mainly retained in the unDRXed regions, whereas the DRXed grains exhibited more randomized orientations after extrusion at 300 °C.

### 3.4. Mechanical Properties of the Extruded Alloys

**Figure 11:**
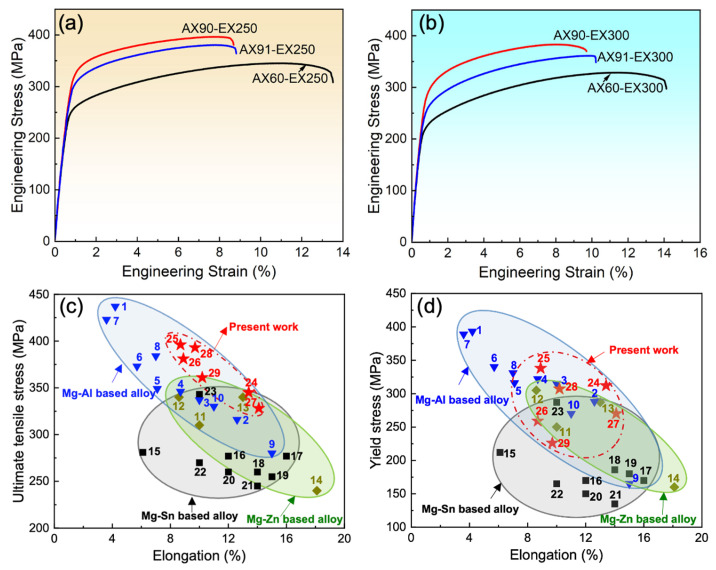
Tensile properties of the extruded Mg-Al-Ca alloys and comparison with reported non-rare-earth Mg alloys. Engineering stress–strain curves of (**a**) AX60-EX250, AX90-EX250, and AX91-EX250 and (**b**) AX60-EX300, AX90-EX300, and AX91-EX300. Strength–ductility plots comparing the present alloys with reported Mg-Al-, Mg-Zn-, and Mg-Sn-based alloys in terms of (**c**) UTS versus elongation and (**d**) YS versus elongation.

Engineering stress–strain curves of the alloys extruded at 250 °C and 300 °C are presented in Figure 11a,b, and the corresponding tensile properties are summarized in Table 2. For the alloys extruded at 250 °C, AX90-EX250 exhibited the highest strength, with a YS of 338 ± 4 MPa and a UTS of 396 MPa, while maintaining an elongation of 8.7 ± 1.6%. AX91-EX250 showed a lower strength than AX90-EX250, with a YS of 312 ± 8 MPa and a UTS of 381 ± 9 MPa, but a similar elongation of 8.9 ± 1.4%. In comparison, AX60-EX250 exhibited a lower YS of 259 ± 6 MPa and a UTS of 345 ± 8 MPa but a higher elongation of 13.4 ± 1.0%. A similar trend was observed for the alloys extruded at 300 °C. AX90-EX300 still showed the highest strength among the three alloys, with a YS of 307 ± 6 MPa, a UTS of 393 ± 7 MPa, and an elongation of 9.7 ± 1.6%, whereas AX91-EX300 exhibited reduced strength with a YS of 270 ± 10 MPa and a UTS of 361 ± 10 MPa. AX60-EX300 showed the lowest strength but the highest ductility, with a YS of 226 ± 7 MPa, a UTS of 328 ± 10 MPa, and an elongation of 14.1 ± 1.7%. Compared with extrusion at 250 °C, extrusion at 300 °C generally decreased the YS but slightly improved the elongation, which is consistent with the coarser and more fully recrystallized microstructures observed at the higher extrusion temperature.

A comparison with reported non-rare-earth Mg alloys is shown in Figure 11c,d, and the corresponding literature data are listed in Table 2. The present Mg-Al-Ca alloys are located in the high-strength region among reported Mg-Al-, Mg-Zn-, and Mg-Sn-based alloys. In particular, AX90-EX250 and AX90-EX300 exhibited a favorable strength–ductility combination, with UTS values close to 400 MPa and elongations of approximately 10%. Compared with many Mg-Zn- and Mg-Sn-based alloys, the present alloys showed a higher YS and UTS. Although some reported Mg-Al-based alloys exhibited comparable or even higher strength, their elongations were generally lower. These comparisons indicate that the AX90 alloy provides a relatively balanced tensile performance among non-rare-earth Mg alloys, especially when processed by extrusion at 250 °C.

## 4. Discussion

### 4.1. Effects of Al and Ca Contents on Microstructure Evolution During Extrusion

The microstructural evolution of the investigated Mg-Al-Ca alloys was closely related to the competitive formation of f-Mg_17_Al_12_, c-Mg_17_Al_12_, and Al_2_Ca during extrusion. As shown in Figure 3 and Figure 7, all extruded alloys exhibited banded deformation-processed microstructures along the ED, but the amount and morphology of the second phases varied with the Al and Ca contents. Quantitative analysis of the BSE images showed that the area fraction of c-Mg_17_Al_12_ increased from approximately 3.9 ± 1.2% in AX60-EX250 to 5.2 ± 1.3% in AX90-EX250 and 5.6 ± 1.4% in AX91-EX250. After extrusion at 300 °C, the corresponding values were approximately 2.0 ± 0.8%, 5.5 ± 0.9%, and 6.4 ± 0.6% in AX60-EX300, AX90-EX300, and AX91-EX300, respectively. The Al_2_Ca phase also showed a composition-dependent variation, with area fractions of approximately 1.4 ± 0.2%, 2.2 ± 0.6%, and 2.5 ± 0.5% in AX60-EX250, AX90-EX250, and AX91-EX250, respectively, and approximately 1.8 ± 0.3%, 1.4 ± 0.5%, and 1.7 ± 0.6% in AX60-EX300, AX90-EX300, and AX91-EX300, respectively.

**Figure 12:**
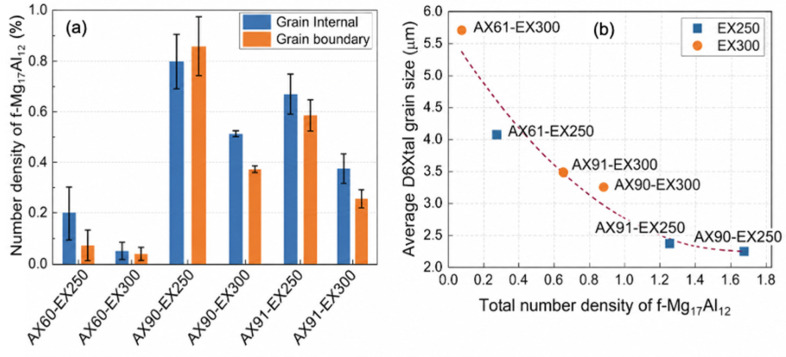
Quantitative analysis of f-Mg_17_Al_12_ particle distribution and its relationship with DRXed grain size in the extruded alloys. (**a**) Number densities of f-Mg_17_Al_12_ particles located in grain interiors and at grain boundaries. (**b**) Relationship between the total number density of f-Mg_17_Al_12_ particles and the average DRXed grain size, where the total number density is calculated by summing the grain-internal and grain-boundary particles.

When the Ca content was fixed at 0.5 wt. %, increasing the Al content from AX60 to AX90 significantly increased the number density of f-Mg_17_Al_12_, as shown in Figure 4 and Figure 8. In AX90, the higher Al content provided sufficient solute for the formation of f-Mg_17_Al_12_, while the relatively low Ca content avoided excessive consumption of Al by Ca-rich intermetallic compounds. The quantitative results in Figure 12 provide further evidence for the role of f-Mg_17_Al_12_ in DRXed grain refinement. As shown in Figure 12a, AX90-EX250 contained the highest number density of f-Mg_17_Al_12_, with 0.805 ± 0.11% located within grains and 0.873 ± 0.16% located at grain boundaries. Correspondingly, AX90-EX250 exhibited the smallest DRXed grain size of 2.27 μm. In contrast, AX60-EX250 contained only 0.277 ± 0.131% f-Mg_17_Al_12_ particles in total, most of which were located within grains, and its DRXed grain size increased to 4.07 μm. AX91-EX250 showed an intermediate total density of 1.260 ± 0.152% and a DRXed grain size of 2.38 μm. A similar relationship was also observed at 300 °C, where AX60-EX300 had the lowest f-Mg_17_Al_12_ density of 0.076 ± 0.025% and the largest DRXed grain size of 5.70 μm, while AX90-EX300 retained a higher total density of 0.892 ± 0.019% and a smaller DRXed grain size of 3.26 μm. The negative correlation in Figure 12b therefore indicates that an increase in f-Mg_17_Al_12_ density was closely associated with the refinement of DRXed grains. The refinement effect of f-Mg_17_Al_12_ is mainly associated with their influence on recrystallization nucleation and grain boundary migration. Particles located near grain boundaries can restrict the migration of recrystallized grain boundaries through the Zener-pinning effect [40], which agrees with the direct observation in Figure 4c, where f-Mg_17_Al_12_ particles are located near recrystallized boundaries. Intragranular f-Mg_17_Al_12_ particles can also introduce local strain gradients and promote dislocation accumulation during hot deformation, thereby providing favorable sites for DRX nucleation through the particle-stimulated nucleation mechanism. Such effects of second-phase particles on recrystallization have been widely discussed in recrystallization theory and observed in Mg alloys containing intermetallic particles [41,42,43]. Therefore, the high density of f-Mg_17_Al_12_ in AX90, especially the large fraction distributed near grain boundaries, contributed to the finer DRXed microstructure by promoting DRX nucleation and retarding grain boundary migration.

Increasing Ca from AX90 to AX91 produced a different effect from increasing the Al content. Although AX90 and AX91 had similar Al contents, AX91 contained fewer f-Mg_17_Al_12_ particles at both extrusion temperatures, together with more Al_2_Ca and coarse second-phase stringers, as shown in Figure 3, Figure 4, Figure 7 and Figure 8. This indicates that increasing the Ca content changes the partitioning of Al between the Mg matrix and Ca-containing intermetallic compounds. Previous studies have shown that Ca addition can promote the formation of Al_2_Ca or Laves phases and that the Ca/Al ratio strongly affects the phase constitution and recrystallization behavior of Mg-Al-Ca alloys [10,11]. In the present AX91 alloy, the increased Ca content likely consumed more Al to form Al_2_Ca, thereby reducing the amount of Al available for f-Mg_17_Al_12_ formation. As a result, the f-Mg_17_Al_12_-assisted pinning effect was weakened compared with that in AX90, even though AX91 contained more Ca-containing coarse second phases.

The texture results in Figure 6 and Figure 10 are also consistent with the DRX behavior controlled by f-Mg_17_Al_12._ The unDRXed regions showed much stronger basal textures, while the DRXed regions exhibited weaker and more dispersed orientations at both extrusion temperatures. This indicates that DRX was responsible for weakening the deformation texture. Since f-Mg_17_Al_12_ particles promoted finer and more extensive DRX, especially in AX90, they indirectly contributed to texture weakening by increasing the fraction of recrystallized grains with more scattered orientations. Therefore, the microstructural evolution during extrusion was governed mainly by the formation and distribution of f-Mg_17_Al_12_, while Ca affected this process by changing Al partitioning and reducing the amount of f-Mg_17_Al_12_ available for grain boundary pinning.

### 4.2. Strengthening and Ductilizing Mechanisms

The tensile results show that AX90 exhibited the highest strength among the three alloys at both extrusion temperatures, whereas AX60 retained higher elongation but lower strength. As summarized in Table 2, AX90-EX250 showed a YS of 338 ± 4 MPa and a UTS of 396 ± 5 MPa, while AX90-EX300 still maintained a high UTS of 393 ± 7 MPa, together with a slightly improved elongation of 9.7 ± 1.6%. In comparison, AX60-EX250 and AX60-EX300 showed lower YS values of 259 ± 6 and 226 ± 7 MPa, respectively, but higher elongations of 13.4 ± 1.0% and 14.1 ± 1.7%, respectively. AX91 exhibited intermediate tensile properties, indicating that increasing the Ca content from AX90 to AX91 did not further enhance the strength. These differences can be understood from the combined effects of grain boundary strengthening, solid-solution strengthening, second-phase strengthening, texture, and deformation compatibility. Grain-boundary strengthening is mainly associated with the DRXed grain size, solid-solution strengthening originates primarily from Al dissolved in the α-Mg matrix, and second-phase strengthening is mainly contributed by finely distributed f-Mg_17_Al_12_ particles. The strengthening contribution from coarse c-Mg_17_Al_12_ and Al_2_Ca stringers was not separately calculated here because these coarse phases are less effective for Orowan strengthening and may also introduce local stress concentrations during tensile deformation.

The grain-boundary strengthening contribution was estimated using the Hall–Petch relationship:

(1) $$\sigma_{GB}=\sigma_{0}+kd^{-1/2}$$

where $\sigma_{0}$ is the friction stress, *k* is the Hall–Petch coefficient, and *d* is the average DRXed grain size. Hall–Petch strengthening has been widely used to describe grain refinement strengthening in Mg alloys [44]. In terms of Al-containing Mg alloys, a relation of $\sigma_{0.2}=62+202d^{-1/2}$ has been reported for randomly textured Mg-Al-Zn alloys [45]. Therefore, $\sigma_{0}=62$ MPa and $k=202\;{\mathrm{MPa}\mu m}^{1/2}$ were used here for a conservative estimation. The calculated $\sigma_{GB}$ values were 162.1, 196.1, and 192.9 MPa for AX60-EX250, AX90-EX250, and AX91-EX250, respectively. When the extrusion temperature increased to 300 °C, grain coarsening reduced $\sigma_{GB}$ to 146.6, 173.9, and 170.1 MPa for AX60-EX300, AX90-EX300, and AX91-EX300, respectively. The stronger grain-boundary strengthening in AX90 was consistent with its finer DRXed grains.

The chemical compositions of the α-Mg matrix in the extruded alloys are listed in Table 3. The Al content dissolved in the matrix increased from 5.51–5.60 at.% in AX60 to 7.10–7.50 at.% in AX90 and AX91. In contrast, only a trace amount of Ca, approximately 0.03–0.05 at.%, was retained in the α-Mg matrix. This indicates that Al was the dominant solute contributing to solid-solution strengthening, whereas the direct strengthening contribution from dissolved Ca was limited.

The solid-solution strengthening contribution was calculated using the multicomponent solid-solution strengthening model proposed by Gypen and Deruyttere [46,47]:

(2) $$\sigma_{ss}={\left(\sum_{i}k_{i}^{1/n}c_{i}\right)}^{n}$$

where $k_{i}$ is the strengthening coefficient of solute element *i*, $c_{i}$ is the solute concentration expressed as an atomic fraction, and *n* is taken as 2/3 [48]. Since the Ca content dissolved in the α-Mg matrix was only 0.03–0.05 at. %, only the contribution from Al was considered. Using $k_{Al}=196$ MPa and n=2/3, the equation can be simplified as

(3) $$\sigma_{ss}=k_{Al}c_{Al}^{2/3}$$

Based on the measured Al contents in the α-Mg matrix, the calculated $\sigma_{ss}$ values were 28.4, 34.2, and 33.6 MPa for AX60-EX250, AX90-EX250, and AX91-EX250, respectively. The corresponding values for AX60-EX300, AX90-EX300, and AX91-EX300 were 28.7, 34.9, and 33.9 MPa, respectively. These results indicate that AX90 and AX91 had slightly stronger solid-solution strengthening than AX60, but the difference was only about 5–6 MPa. Therefore, solid-solution strengthening alone cannot explain the higher YS of AX90.

The strengthening contribution from f-Mg_17_Al_12_ particles was estimated using an Orowan-type mechanism. Since f-Mg_17_Al_12_ particles were finely distributed within grains and near grain boundaries, they could act as non-shearable obstacles to dislocation motion. The Orowan strengthening contribution can be expressed as [49,50]

(4) $$\sigma_{Or}=M\frac{0.4Gb}{\pi \sqrt{1-\nu \lambda }}\ln(\frac{d_{p}}{b})$$

where *M* is the Taylor factor, *G* is the shear modulus of Mg, *b* is the Burgers vector of <a> dislocations, ν is Poisson’s ratio, $d_{p}$ is the average diameter of f-Mg_17_Al_12_, and λ is the inter-particle spacing. The parameters M=3.06, G=17 GPa, b=0.32 nm, and ν=0.35 were used for Mg alloys [49,50,51]. Thus, the equation can be simplified as

(5) $$\sigma_{Or}(\mathrm{MPa})=\frac{2629}{\lambda }\ln(\frac{d_{p}}{0.32})$$

In this calculation, $d_{p}=200$ nm was used as a representative value based on the high-magnification BSE images. The inter-particle spacing was estimated from the total number density $N_{A}$ of f-Mg_17_Al_12_:

(6) $$\lambda =\frac{1000}{\sqrt{N_{A}}}-d_{p}$$

where $N_{A}$ is expressed in ${\mu m}^{-2}$. The calculated $\Delta \sigma_{Or}$ values were 10.0, 29.6, and 24.5 MPa for AX60-EX250, AX90-EX250, and AX91-EX250, respectively. At 300 °C, the values decreased to 4.9, 19.7, and 16.4 MPa for AX60-EX300, AX90-EX300, and AX91-EX300, respectively. The larger Orowan strengthening contribution in AX90 originated from its higher f-Mg_17_Al_12_ number density and smaller inter-particle spacing.

**Figure 13:**
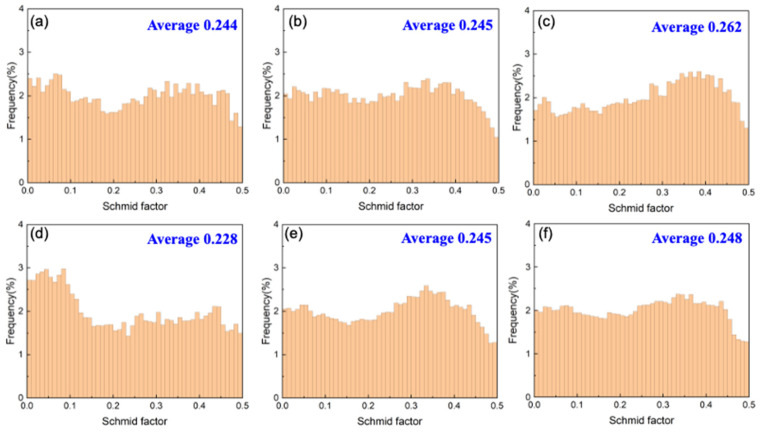
Distributions of the SF of $\{0001\}<11\bar{2}0>$ slip in (**a**) AX60-EX250; (**b**) AX90-EX250; (**c**) AX91-EX250; (**d**) AX60-EX300; (**e**) AX90-EX300; (**f**) AX91-EX300.

The texture effect was evaluated separately based on the average Schmid factor (SF) for basal slip. The distributions of the SF for basal slip are shown in Figure 13. The average SF values were 0.244, 0.245, and 0.262 for AX60-EX250, AX90-EX250, and AX91-EX250, respectively, and 0.228, 0.245, and 0.248 for AX60-EX300, AX90-EX300, and AX91-EX300, respectively. According to Schmid’s law, a lower SF indicates that a higher applied stress is required to activate basal slip. Therefore, texture can influence the yielding behavior of the extruded alloys. However, the variation in SF does not fully follow the measured YS trend. For example, AX60-EX250 and AX90-EX250 showed nearly identical average SF values, whereas AX90-EX250 exhibited a much higher YS. Similarly, AX60-EX300 had the lowest average SF among the alloys extruded at 300 °C but showed the lowest YS. These results indicate that texture affects the yielding behavior, but it was not the dominant factor responsible for the strength difference among the present alloys.

The above strengthening analysis indicates that the difference in the YS among the present alloys is mainly governed by grain-boundary strengthening and f-Mg_17_Al_12_-related Orowan strengthening, while solid-solution strengthening and texture effect play secondary roles. Grain-boundary strengthening provides the largest contribution to the YS because of the fine DRXed grains. More importantly, this contribution is not independent of the second phases. As discussed in Section 4.1, the refinement of DRXed grains is closely related to the presence of f-Mg_17_Al_12_, which promotes DRX nucleation and pins recrystallized grain boundaries. Therefore, the primary strengthening mechanism, namely, grain-boundary strengthening, is indirectly controlled by f-Mg_17_Al_12_-assisted grain refinement.

Compared with AX60, AX90 shows much finer DRXed grains and a higher number density of f-Mg_17_Al_12_ particles at both extrusion temperatures. As a result, AX90 exhibits stronger grain-boundary strengthening and a larger Orowan strengthening contribution. In contrast, the increase in solid-solution strengthening from AX60 to AX90 is limited because the calculated $\sigma_{ss}$ differs by only about 5–6 MPa. This indicates that the higher YS of AX90 is mainly derived from the dual role of f-Mg_17_Al_12_: refining DRXed grains and directly impeding dislocation motion.

Compared with AX90, AX91 has a similar Al content in the α-Mg matrix and therefore a comparable solid-solution strengthening contribution. However, increasing Ca from AX90 to AX91 promotes the formation of coarse Al_2_Ca-containing second phases and reduces the effective density of f-Mg_17_Al_12_. Consequently, both f-Mg_17_Al_12_-assisted grain refinement and Orowan strengthening are weakened in AX91. Although AX91 still maintains a relatively fine DRXed microstructure, the reduced density of f-Mg_17_Al_12_ and the increased amount of coarse Ca-containing intermetallics prevent further improvement in the YS. Therefore, the superior strength of AX90 mainly arises from an optimized second-phase constitution, in which dense f-Mg_17_Al_12_ particles contribute simultaneously to DRX grain refinement and dislocation obstruction, while excessive coarse Al_2_Ca formation is avoided.

The ductility of the present alloys was closely related to matrix deformability and second-phase morphology. Although the Ca content dissolved in the α-Mg matrix was very low, approximately 0.03–0.05 at. %, it could still influence the activation of deformation modes. Previous studies have shown that dilute Ca addition can reduce the difference in critical resolved shear stress between basal and non-basal slip systems, weaken deformation anisotropy, and promote more compatible plastic deformation in Mg alloys [52,53]. In the present alloys, such trace dissolved Ca helped the α-Mg matrix accommodate local strain around f-Mg_17_Al_12_ particles and second-phase/matrix interfaces, which was beneficial for maintaining ductility while strengthening was introduced. This effect was particularly important for AX90, where dense f-Mg_17_Al_12_ particles provided strengthening but remained fine and relatively dispersed, avoiding severe premature cracking.

However, the beneficial effect of Ca should be distinguished from the effect of excessive Ca addition. When the Ca content increased from AX90 to AX91, more Al_2_Ca stringers were formed along the ED. These coarse intermetallic compounds were less effective for Orowan strengthening and could serve as local stress-concentration sites during tensile deformation. Meanwhile, they consumed Al and reduced the formation of f-Mg_17_Al_12_, thereby weakening both grain-boundary strengthening and Orowan strengthening. Therefore, Ca played a composition-dependent role in Mg–Al–Ca alloys: a small amount of Ca dissolved in the matrix may improve deformation compatibility, whereas excessive Ca promotes the formation of coarse Al_2_Ca-containing phases and becomes detrimental to the strength–ductility balance.

These results suggest a practical design strategy for wrought Mg–Al–Ca alloys. The Al content should be sufficient to provide Al solid-solution strengthening and promote a high density of f-Mg_17_Al_12_ particles during extrusion. These f-Mg_17_Al_12_ particles play a dual role in refining DRXed grains and providing Orowan strengthening. In contrast, the Ca content should be carefully controlled at a moderate level. It should be high enough to retain a trace amount of dissolved Ca in the α-Mg matrix and contribute to deformation compatibility, but not so high as to promote excessive Al_2_Ca formation and coarse second-phase stringers. From this viewpoint, AX90 represents the most favorable composition in the present alloy series because it balances Al-driven f-Mg_17_Al_12_ formation, DRX grain refinement, and limited formation of coarse Ca-containing phases, leading to the best strength–ductility combination.

## 5. Conclusions

In this work, three Mg-Al-Ca alloys with different Al and Ca contents were extruded at 250 °C and 300 °C to clarify the roles of Al and Ca in microstructure evolution and mechanical properties. The results showed that Al mainly promoted the formation of f-Mg_17_Al_12_, whereas excessive Ca favored the formation of the Al_2_Ca phase. The main conclusions are as follows:

(1) Increasing the Al content from AX60 to AX90 significantly increased the density of f-Mg_17_Al_12_ particles, leading to finer DRXed grains. AX90-EX250 contained the highest f-Mg_17_Al_12_ density and exhibited the smallest DRXed grain size of 2.27 μm. This refinement was attributed to particle-assisted DRX nucleation and the pinning of recrystallized grain boundaries. In contrast, increasing the Al content from AX90 to AX91 promoted the Al_2_Ca phase, thereby reducing the effective density of f-Mg_17_Al_12_.
(2) AX90 exhibited the best strength–ductility balance among the studied alloys. AX90-EX250 achieved a YS of 338 ± 4 MPa, a UTS of 396 ± 5 MPa, and an elongation of 8.7 ± 1.6%, while AX90-EX300 maintained a high UTS of 393 ± 7 MPa with an improved elongation of 9.7 ± 1.6%. Compared with AX60, the higher strength of AX90 was mainly associated with finer DRXed grains and a higher density of f-Mg_17_Al_12_. Compared with AX91, AX90 benefitted from fewer coarse Ca-containing second phases and more effective f-Mg_17_Al_12_-assisted strengthening.
(3) Strengthening analysis indicated that grain-boundary strengthening was the dominant contribution to the YS, while f-Mg_17_Al_12_-related Orowan strengthening provided an additional contribution. These two strengthening effects were both linked to f-Mg_17_Al_12_, which refined DRXed grains and directly impeded dislocation motion. The ductility was mainly associated with matrix deformability and second-phase morphology. Trace Ca dissolved in the α-Mg matrix may improve deformation compatibility, whereas excessive Ca promotes coarse Al_2_Ca-containing intermetallic compounds and local strain concentration. Therefore, high-strength Mg–Al–Ca extrusion alloys should be designed by promoting dense f-Mg_17_Al_12_ formation while avoiding excessive Ca-induced coarse intermetallic compounds.

## Figures and Tables

**Table 1:** Actual chemical compositions of the AX60, AX90, and AX91 alloys determined by ICP-AES analysis (wt. %).

Sample No.	Al	Ca	Fe	Ni	Cu	Si	Mg
AX60	6.1	0.489	0.004	0.0003	0.0004	0.048	bal.
AX90	8.83	0.491	0.005	0.0004	0.0003	0.041	bal.
AX91	8.91	1.32	0.005	0.0003	0.0004	0.028	bal.

**Table 2:** Tensile properties of the present Mg–Al–Ca alloys and representative non-rare-earth Mg alloys reported in the literature [18,26,27,28,29,30,31,32,33,34,35,36,37,38,39]. The alloy numbers correspond to the data labels used in the strength–ductility plots in Figure 11c,d.

No.	Alloy Compositions	YS (MPa)	UTS (MPa)	Elongation (%)	References
1	Mg-6Al-3Ca-0.3Mn	389	423	3.6	[18]
2	Mg-0.6Al-0.6Ca-0.25Mn	288	316	12.6	[26]
3	Mg-1.1Al-1.1Ca-0.25Mn	315	337	10	[26]
4	Mg-2.3Al-2.3Ca-0.4Mn	322	346	8.7	[26]
5	Mg-3.6Al-3.4Ca-0.4Mn	340	373	5.7	[26]
6	Mg-4.8Al-4.7Ca-0.4Mn	316	349	7.1	[26]
7	Mg-8Al-0.5Zn	393	437	4.2	[27]
8	Mg-8Al-0.5Zn	331	384	7	[28]
9	Mg-6Al-1Zn	165	280	15	[29]
10	Mg-8Al-0.5Zn	270	330	11	[29]
11	Mg-4.5Zn-1.13Ca	250	310	10	[30]
12	Mg-6Zn-1.5Ca	305	340	8.6	[31]
13	Mg-6Zn-0.7Ca-0.2Ce	287	340	13	[32]
14	Mg-2Zn-1.6Ca	160	240	18.1	[33]
15	Mg-6Sn	212	281	6.1	[34]
16	Mg-5Sn	170	277	12	[35]
17	Mg-4Sn	170	277	16	[36]
18	Mg-8Sn-2Zn-2Al	186	260	14	[37]
19	Mg-8Sn-2Zn-2Al	180	255	15	[37]
20	Mg-5Sn-1Zn-1Al	150	260	12	[38]
21	Mg-5Sn-1Zn-3Al	135	245	14	[38]
22	Mg-5Sn-1Zn-5Al	165	270	10	[38]
23	Mg-5Sn-1Si-0.6Ca	287	343	10	[39]
24	AX60-EX250	259 ± 6	345 ± 8	13.4 ± 1.0	—
25	AX90-EX250	338 ± 4	396 ± 5	8.7 ± 1.6	—
26	AX91-EX250	312 ± 8	381 ± 9	8.9 ± 1.4	—
27	AX60-EX300	226 ± 7	328 ± 10	14.1 ± 1.7	—
28	AX90-EX300	307 ± 6	393 ± 7	9.7 ± 1.6	—
29	AX91-EX300	270 ± 10	361 ± 10	10.2 ± 0.9	—

**Table 3:** EDS-measured chemical compositions of the α-Mg matrix in the extruded Mg–Al–Ca alloys.

Alloys	Mg (at.%)	Al (at.%)	Ca (at.%)
AX60-EX250	94.46 ± 0.23	5.5 ± 0.22	0.04 ± 0.01
AX60-EX300	94.35 ± 0.25	5.6 ± 0.24	0.05 ± 0.01
AX90-EX250	92.66 ± 0.30	7.3 ± 0.29	0.04 ± 0.01
AX90-EX300	92.46 ± 0.32	7.5 ± 0.31	0.04 ± 0.01
AX91-EX250	92.86 ± 0.41	7.1 ± 0.40	0.04 ± 0.01
AX91-EX300	94.77 ± 0.56	7.2 ± 0.55	0.03 ± 0.01

## Data Availability

The original contributions presented in this study are included in the article. Further inquiries can be directed to the corresponding authors.
